# Rare mutations in apoptosis related genes *APAF1*, *CASP9*, and *CASP3* contribute to human neural tube defects

**DOI:** 10.1038/s41419-017-0096-2

**Published:** 2018-04-30

**Authors:** Xiangyu Zhou, Weijia Zeng, Huili Li, Haitao Chen, Gang Wei, Xueyan Yang, Ting Zhang, Hongyan Wang

**Affiliations:** 1 Obstetrics & Gynecology Hospital, Institute of Reproduction and Development, State Key Laboratory of Genetic Engineering at School of Life Sciences, Fudan University, 200011 Shanghai, China; 2 Shanghai First Maternity and Infant Hospital, Tongji University School of Medicine, 201204 Shanghai, China; 3 Capital Institute of Pediatrics, 100020 Beijing, China

Dear Editor:

Neural tube defects (NTDs) are common congenital anomalies that occur because of the failure of neural tube closure in early embryogenesis. More than 250 NTD causative genes have been reported in mouse models. This suggests the involvement of distinct molecular mechanisms in NTD etiology, including the apoptosis pathway^1^. *APAF1*, *CASP9*, and *CASP3* knockout mice display typical NTD phenotypes including severe craniofacial malformations and exencephaly^2–5^. Genes identified using mouse models have been explored as candidates in human NTDs and corresponding analyses of risk association. However, examinations of human populations have not provided persuasive genetic evidence to support a role for the apoptosis pathway in human NTDs.

To further understand the etiology of human NTDs, we performed next generation capture target sequencing of 3 apoptosis related genes in 352 NTD patients and 224 controls. We identified 14 non-synonymous candidate variants (protein-altering, minor allele frequency <1%) in three apoptosis related genes *CASP9*, *APAF1,* and *CASP3*. All these variants were case-specific variants that only exist in 352 NTD samples but not in any of 224 controls (Fig. 1a). 10 of 14 candidate variants have no records in 1000 Genomes (Fig. S1, Table S1). In addition, we also identified 2 candidate variants in three genes in 224 controls. There is a significant enrichment of candidate variants in NTDs (14/352) vs. controls (2/224) by using fisher’s exact test (*p* = 0.03524). Subsequent sequence alignment of 14 candidate variants in NTDs suggested that *CASP9* R180C (p.Arg180Cys), *CASP3* Q217H (Gln217His), and *APAF1* P335R (Pro335Arg) were extremely conserved in different species and caspase sub-family members (Fig. 1b). Notably, all three variants, located in the protein binding sites, were uniformly predicted to disrupt protein function based on five different software-based algorithms including SIFT, Poly-phen2, Mutation-taster, Mutation-assessor and Provean (Table S1).

**Fig. 1:**
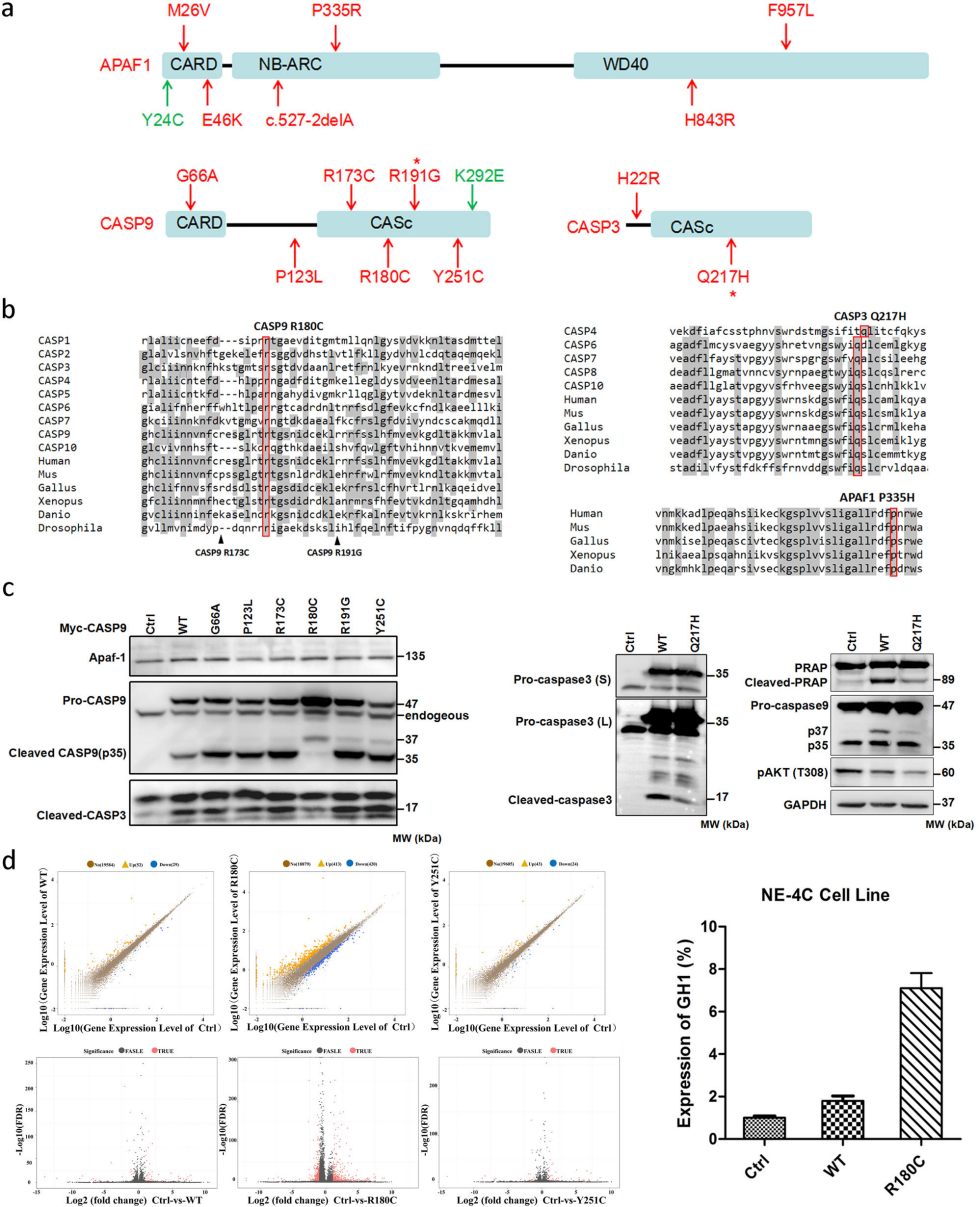
Rare mutations in *APAF1*, *CASP9*, and *CASP3* contribute to human neural tube defects. **a** Illustration of *APAF1*, *CASP9*, *CASP3* protein structure with non-synonymous variants identified in 352 NTD cohorts (red arrow) and 224 control group (green arrow). Asterisk indicates the recurrent mutations that affect more than 2 cohorts. **b** Representative results of sequence alignment including *CASP9* R180C, *CASP3* Q217H, and *APAF1* P335R in different species and caspase subfamily members. Mus, house mouse. Gallus, chicken. Xenopus, clawed frog. Danio, Zebra fish. Drosophila, fruit fly. **c** Rare mutations in *CASP9*, especially R180C totally blocked procaspase-9 cleavage in transfected 293T cells, as a result, cleaved-CASP3 was reduced. (Left). *CASP3* Q217H impairs spontaneous cleavage of CASP3, as well as downstream PARP. The phosphorylation of Akt at Thr308 site and release of p37 subunit from CASP9 were reduced by Q217H when compared with wild type CASP3 (Right). GAPDH served as a loading control. S, short exposure; L, long time exposure. **d** Volcano plot of control vs WT or mutant CASP9. Significant differences were detected in R180C samples compared to wild type and Y251C samples (Left). The log2 fold change between the treatment means is plotted on the horizontal axis. The −log10 FDR (adjusted *p*-values by Fisher’s test) is plotted on the vertical axis. Black points are not statistically significant, and red points are significant at *p* < 0.01 and fold-change>2. The representative differential expressed gene GH1 identified by RNA-seq was confirmed in the NE-4C cell line by qPCR (Right)

We next performed functional analyses on these candidate variants. Especially, using previously well-defined *CASP9* C287A (active site) and D315A (cleavage site) mutations as positive controls^6^, we found that rare mutation in *CASP9*, especially R180C totally abolished spontaneous cleavage of CASP9 whereas *CASP9* K292E identified from controls has no effect on procaspase9 cleavage (Fig. 1c and S2). Additionally, cleaved CASP3 and PARP, downstream CASP9 targets, were markedly reduced by R180C. In addition, CASP3 Q217H severely affected the spontaneous cleavage of CASP3 and PARP. (Fig. 1c). Interestingly, we found phosphorylation of AKT (T308) and release of p37 subunit from procaspase-9 were also reduced by Q217H. Subsequent analysis of protein interaction using the CheckMate Mammalian Two-Hybrid System indicated that co-transfection of CASP9 (WT) and APAF1 activated reporter activity 2.13-fold. However, co-transfection of CASP9 (R180C) with APAF1 did not activate the reporter, indicating that interaction with APAF1 is disrupted in CASP9 (R180C), as well as CASP9 (R191G) (Fig. S3a). Furthermore, we found that APAF1 M26V significantly impaired the protein interaction with CASP9 (Fig. S3b).

We also performed RNA-seq on HEK cells overexpressing recombinant CASP9-Myc proteins to identify differentially expressed genes (DEG). Overall, 81 and 67 DEGs were identified in HEK cells transfected with WT CASP9 and CASP9 (Y251C), respectively, when compared to the control sample (Fig. 1d). Unexpectedly, 833 DEGs were detected in HEK cells transfected with CASP9 (R180C). Of note, both GH1 and GH2, which play critical roles in growth control, were dramatically up-regulated by over-expression of CASP9 (R180C). To further confirm GH1/2 act as regulatory targets by CASP9 R180C in neural derived cells, we performed RT-qPCR using cDNA derived from NE-4C (mouse embryonic neuroectodermal stem cells) that transfected with WT and R180C CASP9. We found that expression of GH1 and GH2 were significantly up-regulated in NE4C cells (Fig. 1d). Protein structure predictions found amino acid substitution from Arg to Cys at 180 position destroyed two hydrogen that supposed to recognize the Arg, which further cause weak affinity with substrates. Consequently, although lacking data from neural-derived cells, we found overpression of R180C lost the inhibitory role on cell proliferation in HEK 293T cells (Fig. S4).

In the ExAC database, a total 615 non-synonymous variants (MAF < 1%) including 57 LoF variants and 558 missense variants were identified in 60,706 unrelated individuals for three genes. Despite all this, the frequencies of non-synonymous mutations identified in three genes in NTD patients (14/352) were significantly higher than that in ExAC database (615/60706) by using fisher’s exact test (*p*-value = 2.17e-5). Furthermore, the distribution of 57 LoF variants identified from ExAC, especailly 41 singletion LoF variants, were markedly varying in different ethnicities(Fig. S5). No singletion high-confidence LoF variants was derived from East Asian population. In this study, we identified a new singleton LoF variant in *APAF1* in NTDs patients. Although the etiology of NTDs in human is multifactorial^7^, our results strongly suggest that rare mutations in apoptosis-related genes including *CASP9*, *APAF1*, and *CASP3* contribute to etiology of neural tube defects in Han Chinese population.

## Electronic supplementary material

Supplementary files

Supplementary Figure S1

Supplementary Figure S2

Supplementary Figure S3

Supplementary Figure S4

Supplementary Figure S5
