# Supplementary material for: Rare mutations in apoptosis related genes APAF1, CASP9, and CASP3 contribute to human neural tube defects
Source: Cell Death Dis. 2018 Apr 30;9(2):43. doi: 10.1038/s41419-017-0096-2 (PMC5833651; doi:10.1038/s41419-017-0096-2)
Supplement: Supplementary file 1 — Supplementary files [file 41419_2017_96_MOESM1_ESM.doc]

**Supplementary Files**

**Methods**

The basic information of subjects in this study has been described previously [1]. NTD samples were collected from aborted fetuses and children with spina bifida who were less than 10 years old in the period from the 1990s to the 2010s. 352 NTD samples (51.4% female, 38.1% male, 10.5% unknown) were from Shanxi (270), Liaoning (28), Heilongjiang (15), Suzhou (14), and Tianjin (25) provinces. Tissue samples from aborted fetuses with severe rostral NTDs, such as craniorachischisis or anencephaly, accounted for 309 of the 352 cases. The rest of 43 NTD samples were children under the age of 10 with spina bifida from whom blood samples were collected. The 224 controls (58.9% female, 40.6% male, 0.5% unknown) were ethnically and gender-matched unrelated healthy volunteers recruited from Shanxi province and Shanghai, China.

Written informed consent for study participation was obtained prior to commencement. All the procedures in this study were approved by the Medical Ethics Committee of Fudan University and relevant local ethics committees prior to the commencement of the study.

**Target-capture sequencing**

We performed next-generation target-capture sequencing on 284 candidate genes (UTR and coding region) in both 352 patients and 224 control groups using the Agilent SureSelect XT Custom enrichment system. We have described the detailed procedures for library building and target enrichment previously [1]. Briefly, sequencing was performed on an Illumina HiSeq2000 DNA sequencer (version 3, Illumina, Inc.). The fastq files obtained were mapped to hg19 reference sequence using a Burrows-Wheeler Aligner's Smith-Waterman Alignment (BWA-SW). Variants (SNVs and Indels) were annotated with information from Ensemble release 79 using the Variant Effect Predictor based on the hg19/GRCh37 database.

In this study, we mainly focused on protein altering variants (missense, stop, gain or loss, frame shift, and splicing) in *CASP9*, *APAF1* and *CASP3*. Variants with MAF (minor allele frequency) > 1% in the 1000 Genomes database were filtered out. Missense mutations were evaluated using SIFT and PolyPhen-2 for function prediction.

**Immunoblot Analysis**

HEK 293T cells were transfected with wildtype and mutant *CASP9* or *CASP3*  using Lipofectamine 3000 (ThermoFisher). After 36 hours, cells were lysed in RIPA Buffer (Sangon Biotech) supplemented with a 1:200 dilution of protein inhibitor (Sangon Biotech). Following electrophoresis and membrane transfer, protein were probed with one of the following primary antibodies in either 5% non-fat milk or 5% BSA: Apoptosis Antibody Sampler Kit (#9915), anti-APAF1 (#8969), anti-pAKT (#4056) were from CST(Cell Signaling Technology); as well as anti-GAPDH (G9545; Sigma-Aldrich), and anti-Myc (TA150121; Origene). After incubation with secondary HRP-antibodies (either anti-rabbit [7074; CST] or anti-mouse [8270; Sigma-Aldrich]), chemiluminescent detection was performed using ECL1/2 (PC198506/1859701; ThermoFisher). Images were obtained using a Tanon-5200 Chemiluminescent Imaging System (Tanon Science and Technology C., Ltd., Shanghai, China)

**Plasmid Construction**

Myc-tagged human *CASP9* was purchased from Origene Technologies. The QuickChange Site-Directed Mutagenesis Kit (Stratagene, Agilent) was used to generate mutant *CASP9* or *CASP3*. For protein interaction assay, CASP9 ORF was subcloned into the pBIND vector between BamHI and NotI sites to proceed CheckMate Mammalian Two-Hybrid Assay. Flag-tagged human APAF1 was purchased from Vigene Biosciences (CH894566), then a shortened APAF1 fragment (0/+1521bp, 507aa) contains Caspase-9 recruitment domain was subcloned into pACT vector between BamHI and NotI sites to proceed CheckMate Mammalian Two-Hybrid Assay. pGL5 vector was purchased from Promega Corporation.

**Microscopy**

HEK cells were plated in 12-well plates and transiently transfected with wild type and mutant CASP9 to overexpress recombinant protein using Lipofectamine 3000 (Thermo Fisher). Cells transfected with empty pCMV-6 GFP-tagged vector served as an indicator to ensure the transfection efficiency above 80%. After 36 h, CellMask™ Plasma Membrane Stains (Thermo Fisher Ltd) were used to label the plasma membrane. Cells were fixed in 4% paraformaldehyde for 10 min and washed in phosphate buffered saline three times. Cells were then stained by using Hoechst 33258 (100 ul/well) for 10 min at room temperature and then photographed under a fluorescence microscope.

**Cell proliferation**

The number of viable cells in the proliferation assay was determined using the CellTiter 96AQueous One Solution Cell Proliferation Assay (MTS) Kit (Promega Ltd) following the standard protocol. HEK 293T cells were transfected with wildtype and mutant *CASP9* using Lipofectamine 3000 (ThermoFisher). After 36 hours, assays were performed by adding the indicated amount of the CellTiter 96®AQueous One Solution reagent directly to the culture wells, incubating for 1 hour, and recording absorbance at 490 nm using a 96-well plate reader.

**RNA-SEQ**

RNA sequencing was performed at BGI Co.,Ltd by using BGISEQ-500 system. Briefly, 293T were transfected with Myc-tagged wild type, R180C and Y251C *CASP9* to overexpress recommbinant protein using Lipofectamine 3000 (Thermo Fisher). After 36 hrs, cells were collected to perform total RNA isolation. The cDNA library was prepared with 1 ug total RNA for each sample and sequencing was performed according to standard manufacture’s protocol from BGI in the previous study [2]. The average clean reads number of three samples was 24.02 million and average gene mapping ratio is 84.97%. Gene expression level was calculated by RPKM (Reads Per Kilobase of exon model per Million mapped reads) according to the provided standard formula. Genes with expression fold-change <2 were excluded from subsequent analyses.

**Statistics**

Differences in variant frequencies between case subjectes and controls/ExAC were calculated by using Fisher exact test when 25% of the cells have expected counts less than 5. Fisher exact test was done by SAS 9.3 (SAS Instiute). Student’s two-tailed unpaired *t*-tests were used to determine statistical significance of differences between CASP9 wild type and mutant using GraphPad Prism software v. 5.01 (GraphPad Software, Inc., La Jolla, CA, USA). All data are presented as individual samples and mean± SEM. Differences were considered to be significant when P values were < 0.05.

**References**

1. Qiao X, Liu Y, Li P, et al. Genetic analysis of rare coding mutations of CELSR1–3 in congenital heart and neural tube defects in Chinese people[J]. Clinical Science, 2016, 130(24): 2329-2340.
2. Fehlmann T, Reinheimer S, Geng C, et al. cPAS-based sequencing on the BGISEQ-500 to explore small non-coding RNAs[J]. Clinical Epigenetics, 2016, 8(1): 123.

**Supplementary Table**

Table S1. 14 case-specific non-synonymous mutations were identified in apoptosis related genes including *APAF1*, *CASP9* and *CASP3* in the NTD patients.

| Gene | Location | AA | No. in  Case | | Domain | SIFT | Poly-  phen2 | Mutation  Assessor | Mutation  Taster | Provean | ExAC | 1000G | Protein  Binding  Site |
| --- | --- | --- | --- | --- | --- | --- | --- | --- | --- | --- | --- | --- | --- |
| APAF1 | c.76A>G | p.M26V | | 1 | CARD | √ | × | √ | √ | × | 0 | 0 | - |
| APAF1 | c.136G>A | p.E46K | | 1 | CARD | × | × | × | × | × | 1 | 1 | - |
| APAF1 | c.517-2delA | - | | 1 |  | - | - | - | - | - | 0 | 0 | n.a |
| APAF1 | c.1004C>G | p.P335R | | 1 | NA-ARC | √ | √ | √ | √ | √ | 0 | 0 | + |
| APAF1 | c.2528A>G | p.H843R | | 1 | WD40 | √ | √ | × | √ | √ | 0 | 0 | - |
| APAF1 | c.2869T>C | p.F957L | | 1 | WD40 | × | × | × | × | × | 0 | 0 | - |
| CASP3 | c.651G>T | p.Q217H | | 2 | CASc | √ | √ | √ | √ | √ | 5 | 0 | + |
| CASP3 | c.65A>G | p.H22R | | 1 | / | × | √ | √ | × | × | 211 | 9 | - |
| CASP9 | c.752A>G | p.Y251C | | 1 | CASc | × | √ | √ | √ | √ | 10 | 1 | + |
| CASP9 | c.517C>T | p.R173C | | 1 | CASc | × | √ | √ | × | × | 4 | 0 | + |
| CASP9 | c.197G>C | p.G66A | | 1 | CARD | √ | √ | √ | √ | √ | 0 | 0 | - |
| CASP9 | c.571C>G | p.R191G | | 4 | CASc | √ | × | × | × | √ | 2 | 0 | - |
| CASP9 | c.538C>T | p.R180C | | 1 | CASc | √ | √ | √ | √ | √ | 2 | 0 | + |
| CASP9 | c.368C>T | p.P123L | | 1 | / | √ | √ | × | √ | √ | 26 | 2 | - |

AA, amino acid

Five software package including SIFT, Poly-phen2, Mutationassessor, MutationTaster and Provean was used to assess the effect of variants on protein function. √ indicates damaging and × indicates benign.

The number of mutant alleles exist in ExAC or 1000 Genomes were displayed.

Protein binding Site was determined based on data from NCBI Structure or predictions from MutationAssessor. + indicated Positive and - indicated Negative.

**Supplementary Figure Legends**

**Figure S1**. Sanger sequencing of non-synonymous variants that have no frequency records in the 1000 Genomes Database.

**Figure S2.** (a) Rare mutations in *CASP9* disrupt the spontaneous cleavage of PARP and reduce the phosphorylation of Akt at Thr308 site while phosphorylation of ERK1/2 was not affected. GAPDH and APAF1 served as a loading control. (b) Two mutations from previous study *CASP9* C287A (active site) and D315A (cleavage site) served as positive controls. (c) *CASP9* K292E (green arrow) that identified from controls has no significant effect on spontaneous cleavage of CASP9 .

**Figure S3**. (a and b) Rare mutations in *CASP9* or *APAF1* impairs the protein interaction with each other. pBIND-CASP9 and pACT-APAF1 were co-transfected into HEK cells with the pGL4.3 Vector as indicated. Cells were incubated for 48 hours and then lysed for the Dual-Luciferase^TM^ Reporter Assay. Cells transfected with pBIND-empty vector served as a negative control. Three independent experiments were performed, and each sample was repeated in triplicate (***p < 0.001, *p < 0.05). (c) Protein structure predictions suggest substitute the Arg180 with Cys in CASP9 will abolish the pivotal hydrogen bonds with the aspatic acid which further lead to the confirmation change of activation loop to the inactive status. This prediction was based on a published crystal structure data (PDB code:1JXQ) in which active and inactive status of CASP9 conformation were determined by a activation loop.

**Figure S4**. (a) Overexpression of CASP9 R180C has no inhibitory effect on HEK cell proliferation when compared to wild type and Y251C CASP9. CellMask Plasma membrane stains and Hoechst 33258 were used to label the cell plasma membrane and nucleus, respectively. (b) Cell viability assay revealed impaired proliferative response caused by R180C by using CellTiter 96AQueous Kit.

**Figure S5**. (a) Distribution of 41 singleton LoF variants of three genes in different population including European, South Asian, African, East Asian, Latino, Finnish and Other. (b) Distribution of 107 mutant alleles hit by 57 LoF variants (MAF<1%) of three genes in different population.
