# Supplementary figures and images for: Rare mutations in apoptosis related genes APAF1, CASP9, and CASP3 contribute to human neural tube defects
Source: Cell Death Dis. 2018 Apr 30;9(2):43. doi: 10.1038/s41419-017-0096-2 (PMC5833651; doi:10.1038/s41419-017-0096-2)

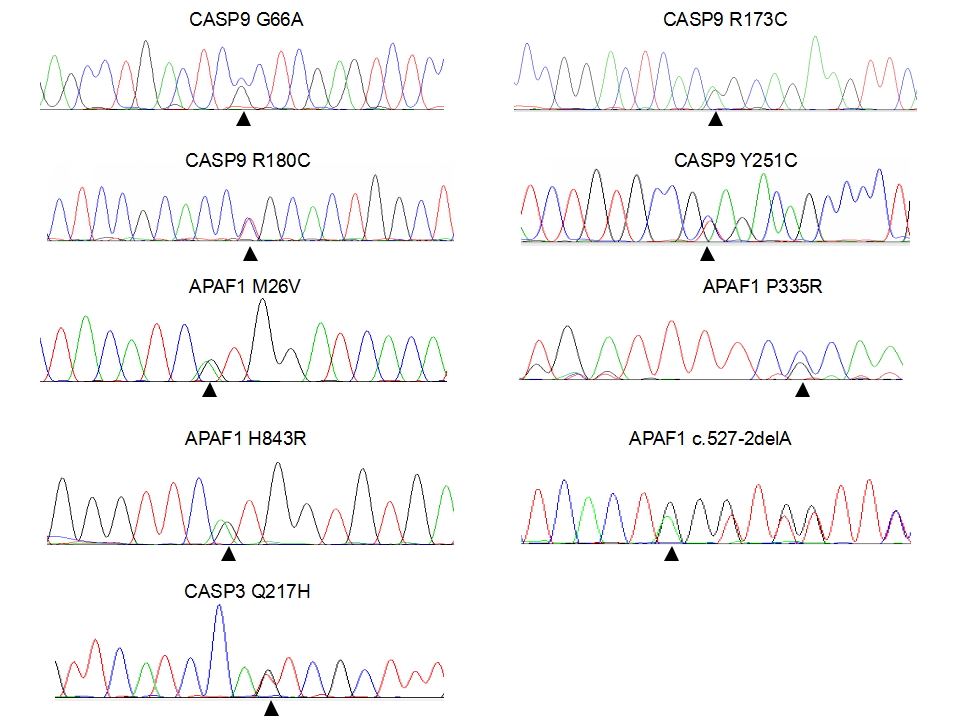

Supplement: Supplementary file 2 — Supplementary Figure S1 [file 41419_2017_96_MOESM2_ESM.tif]

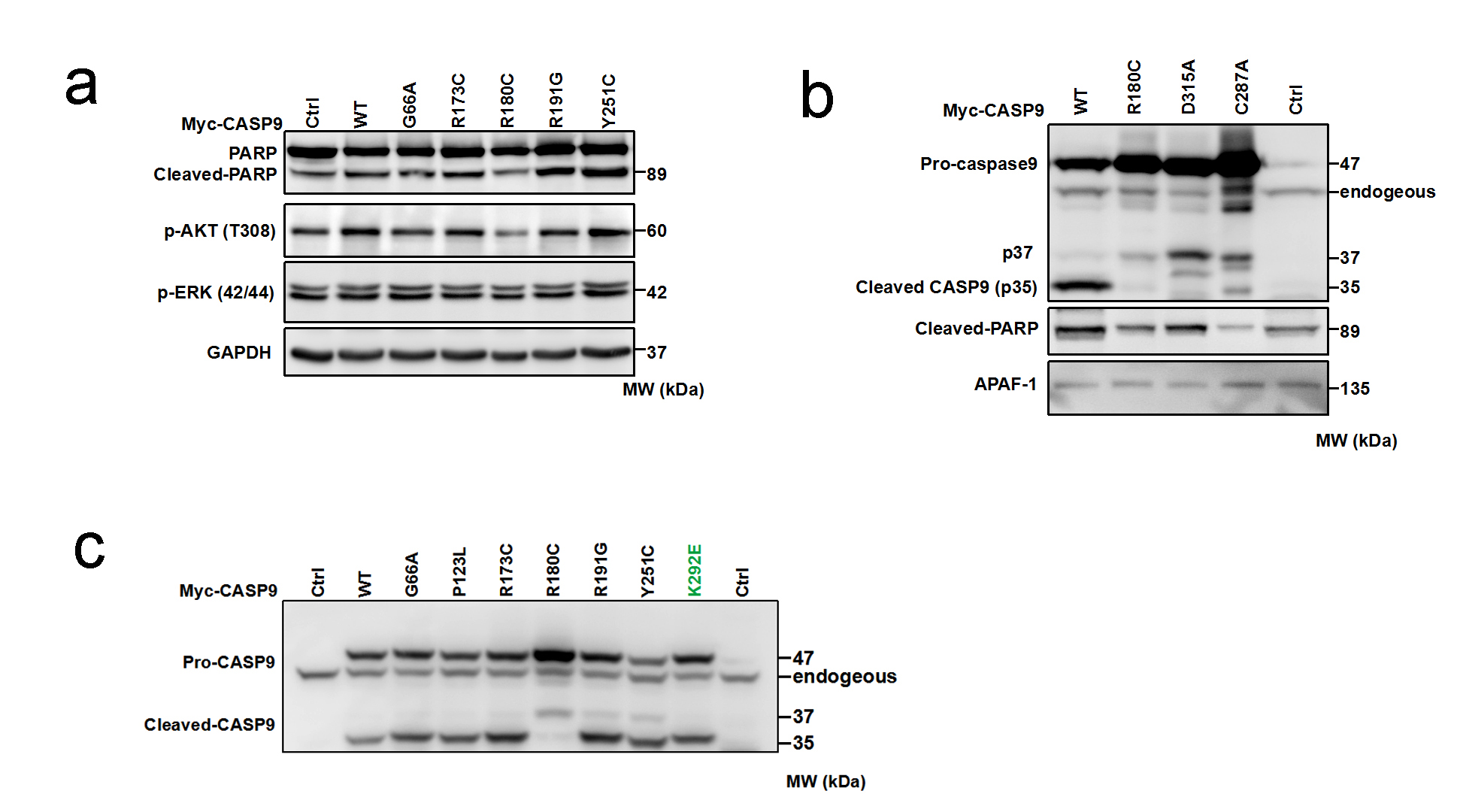

Supplement: Supplementary file 3 — Supplementary Figure S2 [file 41419_2017_96_MOESM3_ESM.jpg]

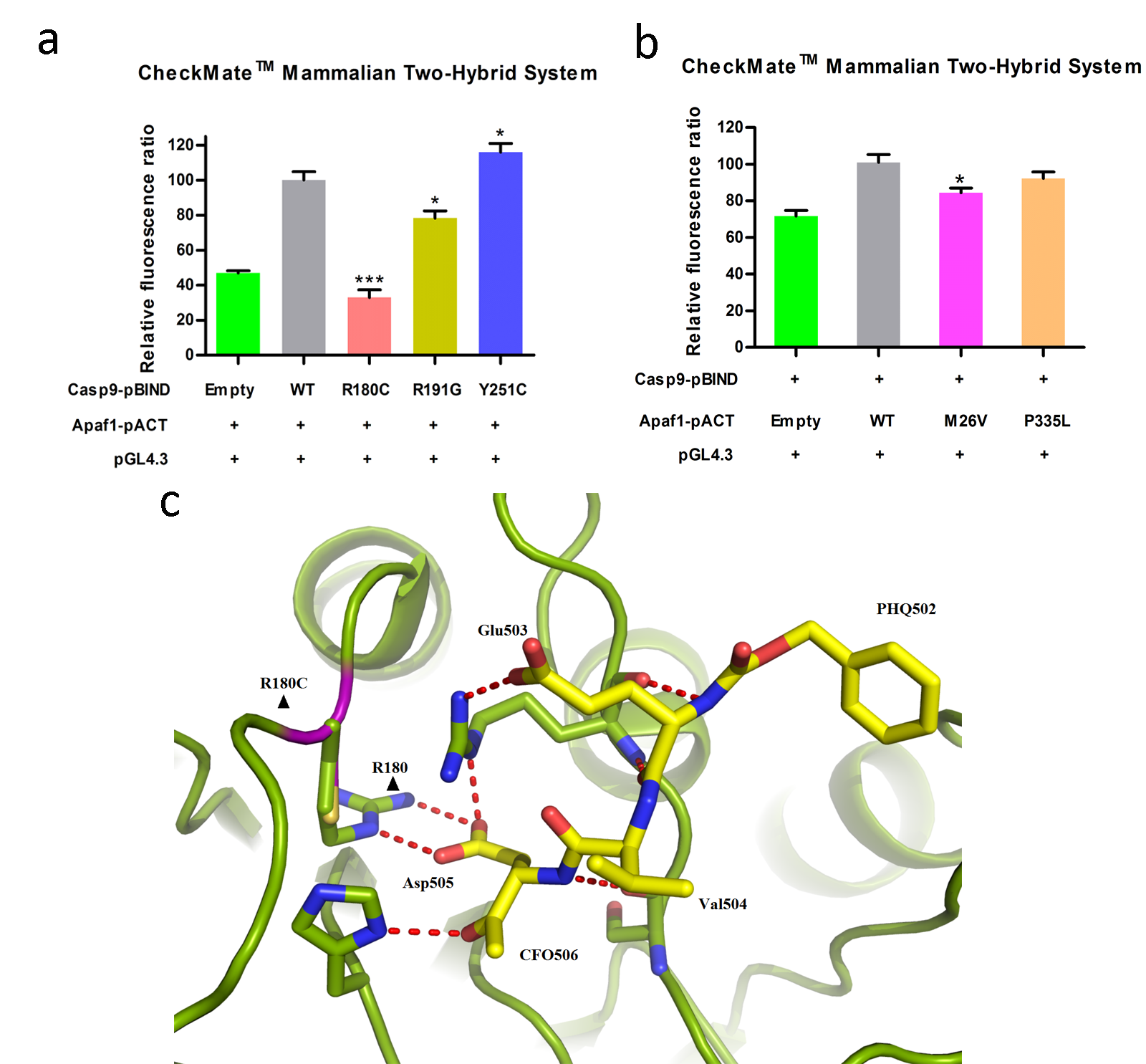

Supplement: Supplementary file 4 — Supplementary Figure S3 [file 41419_2017_96_MOESM4_ESM.tif]

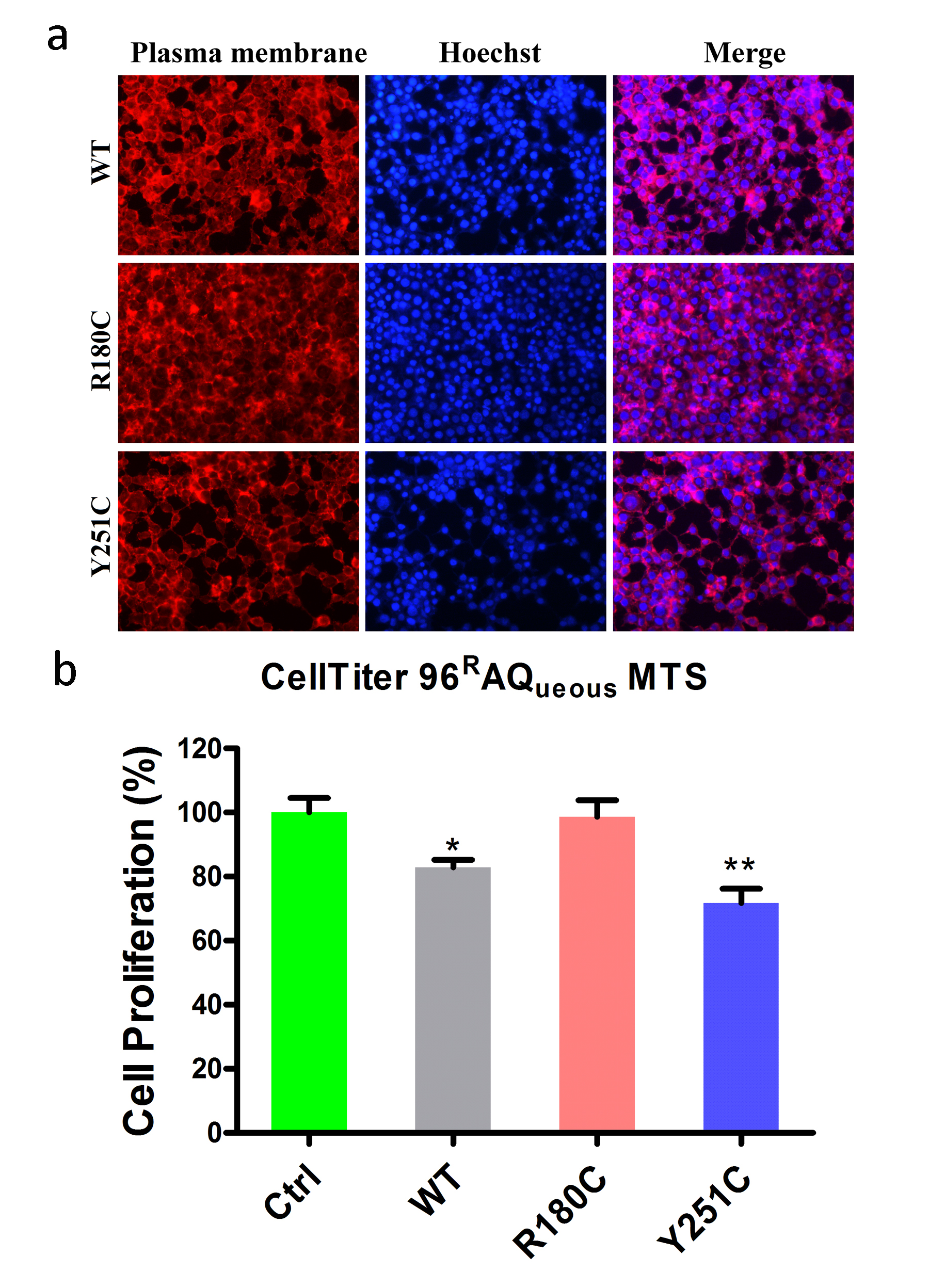

Supplement: Supplementary file 5 — Supplementary Figure S4 [file 41419_2017_96_MOESM5_ESM.jpg]

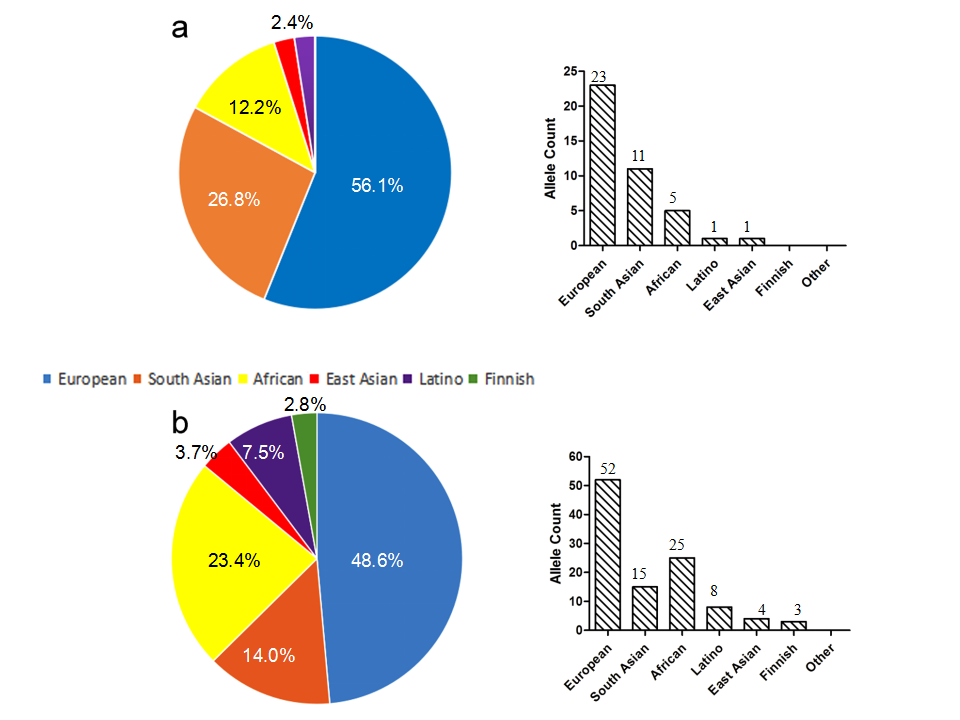

Supplement: Supplementary file 6 — Supplementary Figure S5 [file 41419_2017_96_MOESM6_ESM.tif]
